# Knowledge, Attitudes, and Preparedness Regarding Marburg Virus Disease Among Healthcare Workers in Awi Zone Public Hospitals, Northwest Ethiopia: A Multicenter Cross-Sectional Study

**DOI:** 10.3390/tropicalmed11050125

**Published:** 2026-05-06

**Authors:** Ayenew Genet Kebede, Zewdu Bishaw Aynalem, Aragaw Egziabherfenta Tadele, Belachew Tegegne, Asrat Yazew, Betelhem Mekonnen Alem, Lalem Tilahun, Tamene Fetene Terefe, Getachew Amare, Atsedemariam Andualem, Sewunet Ademe, Yonas Wondie

**Affiliations:** 1 Department of Nursing, College of Medicine and Health Sciences, Injibara University, Injibara P.O. Box 40, Ethiopia; zest7@yahoo.com (Z.B.A.); yoniaragaw21@gmail.com (A.E.T.); beletegegne@gmail.com (B.T.); asratya16@gmail.com (A.Y.); betelhemmekonnen98@gmail.com (B.M.A.); atsede010@gmail.com (A.A.); sewunet.ademe@gmail.com (S.A.); yonasmelkamu88@gmail.com (Y.W.); 2 Department of Pediatrics and Child Health Nursing, College of Medicine and Health Sciences, Injibara University, Injibara P.O. Box 40, Ethiopia; lalemtilahun9@gmail.com (L.T.); tamenefet732@gmail.com (T.F.T.); 3 Department of Midwifery, College of Medicine and Health Sciences, Injibara University, Injibara P.O. Box 40, Ethiopia; getachewamare79@gmail.com

**Keywords:** Marburg virus disease, knowledge, attitude, preparedness, healthcare workers, Ethiopia

## Abstract

Background: An outbreak of Marburg virus in Jinka Town, Southern Ethiopia, has raised significant concern regarding the potential spreading of disease throughout the country. Healthcare workers play a crucial role in early prevention and control of such an outbreak. However, the knowledge, attitudes, and preparedness of healthcare workers regarding Marburg virus disease have not been assessed yet, despite these factors being critical for early prevention and control of an outbreak. This study aimed to assess the knowledge, attitude, and preparedness regarding Marburg virus disease among healthcare workers in Awi Zone public hospitals, northwest Ethiopia, in 2026. Methods: An institutional-based cross-sectional study was conducted among healthcare workers in Awi Zone public hospitals from 26 December 2025 to 10 January 2026. A simple random sampling technique was used to select 394 participants. The data were collected using a pre-tested, structured self-administered questionnaire. The data were entered into Epi-data version 4.6 and analyzed using SPSS version 25. Results: A total of 394 healthcare workers participated in this study. The mean age of the participants was 32.9 ± 4.87 years. The study revealed that 47.7% (95% CI: 42.78–52.62%) and 61.2% (95% CI: 56.4–66%) of participants had good knowledge and a positive attitude towards Marburg virus disease, respectively. However, only 20.3% (95% CI: 16.34–24.26%) demonstrated good preparedness for the Marburg virus outbreak. Conclusions: The study revealed that the majority of healthcare workers had positive attitudes and suboptimal knowledge but critically low preparedness regarding Marburg virus disease prevention and control in Awi Zone public hospitals, northwest Ethiopia. Hence, healthcare workers who are frontline staff for outbreak prevention and control, Awi zone health departments and hospital administrators should be provided with targeted training preparation, training for implementing emergency preparedness plans, and essential infection prevention protocols to improve readiness for a potential outbreak.

## 1. Introduction

Marburg virus (MARV) disease is a highly virulent viral hemorrhagic fever in humans caused by the Marburg virus which belongs to the Filoviridae family [1]. Since its discovery in 1967, MVD has caused sporadic outbreaks with fatality rates ranging from 24% to 88%, depending on the viral strain and the context of the outbreak [2]. The genus Marburg comprises a single species, Marburg virus, which contains two different viral entities: Marburg virus (MARV) and Ravn virus (RAVV) [3]. Clinically, MVD presents with non-specific symptoms such as an abrupt onset of high-grade fever, severe malaise, vomiting, and profound hemorrhagic manifestations, and multi-organ failure. This clinical similarity to other endemic diseases, such as malaria and typhoid fever, often complicates early diagnosis and underscores the critical need for high clinical suspicion among frontline healthcare workers (HCWs) [2]. The virus is of zoonotic origin and is transmitted to people from fruit bats (Rousettus aegyptiacus) and spreads among humans through direct contact with the blood, secretions, organs, or other bodily fluids of infected individuals, and with surfaces and materials contaminated with these substances [4].

The global health community remains on high alert for MVD due to its epidemic potential, as demonstrated by recent outbreaks in Ghana Guinea, and a significant, prolonged outbreak in Equatorial Guinea and Tanzania in 2023 [5]. Ethiopia declared an outbreak of Marburg virus disease (MVD) on 14 November 2025 after the Ethiopian Public Health Institute detected and genomically confirmed the virus in samples taken from suspected cases in the southern region, Jinka town [6].

There are currently no approved vaccines or antiviral treatments; management is purely supportive, making infection prevention and control (IPC) the cornerstone of the response [7]. Preventive measures against Marburg virus infection are not well defined, as transmission from wildlife to humans remains an area of ongoing research. However, avoiding fruit bats and sick non-human primates in central Africa is one way to protect against infection. Early detection through improved surveillance and diagnostics, robust public health systems, and rapid response mechanisms was identified as a crucial component [7].

Marburg virus disease remains a major public health concern due to its high transmissibility, severe clinical course, and high mortality rates [8,9]. Outbreaks can spread rapidly within communities, leading to significant loss of life and placing substantial strain on healthcare systems and national economies [10]. A well-prepared, knowledgeable, and alert healthcare workforce constitutes the first line of defense against Marburg virus outbreaks [11]. However, data from all over Africa indicates that a large number of medical facilities are woefully unprepared [12,13]. Research conducted in high-risk nations has repeatedly revealed significant gaps in medical professionals’ understanding of Marburg virus disease (MVD) symptoms and transmission. There is a lack of confidence regarding institutional preparedness, and healthcare workers have received very little formal education [13]. These gaps have all been linked to an increase in levels of fear, anxiety, and stigmatization [11]. Gaps between knowledge, attitudes, and practices may have serious consequences [14]. They can cause delays in diagnosis, refusal to treat patients, breaches in infection prevention and control, and ultimately, hospital transmission that worsens an outbreak [15].

Ethiopia’s healthcare system faces a dual burden of communicable and non-communicable diseases, and its vulnerability to outbreaks of high-consequence pathogens has been demonstrated by recent epidemics. On 14 November 2025, the Ethiopian Public Health Institute genetically confirmed an outbreak of Marburg virus disease in samples from suspected cases in Jinka Town, southern Ethiopia [6]. This confirmation has placed urgent demands on healthcare workers (HCWs) for effective early prevention, rapid detection, and prompt clinical management to contain the outbreak. The existing literature often treats HCWs as a monolith, failing to account for regional variations in resource allocation and training exposure. Conducting a targeted assessment in the Awi Zone is critical to establishing a baseline of preparedness and to inform the development of context-specific interventions. Therefore, this study aimed to assess the knowledge, attitude, and preparedness regarding Marburg virus disease among HCWs in Awi Zone public hospitals, northwest Ethiopia, in 2026.

## 2. Methods and Materials

### 2.1. Study Design, Period, and Setting

A descriptive cross-sectional study was conducted from 26 December 2025 to 10 January 2026 among healthcare workers working in Awi Zone Public hospitals, northwest Ethiopia. There are five public hospitals in the Awi Zone: Injibara General Hospital, Agew Gimjabet Primary Hospital, Dangila Primary Hospital, Chagni Primary Hospital, and Jawi Primary Hospital. Injibara General Hospital is located in Injibara town, which is 114 and 450 km from Bahir Dar (the capital city of the Amhara region) and Addis Ababa, the capital city of Ethiopia, respectively. The Agew Gimjabet, Dangila, Chagni, and Jawi primary hospitals are located 19.2 km, 36.3 km, and 83.8 km from Injibara Town respectively. These hospitals provide outpatient, inpatient, emergency, medical, and surgical healthcare services for more than 1.3 million of the population. About 856 healthcare workers work in these five hospitals.

### 2.2. Source and Study Population

The source population consisted of all healthcare workers who were working in Awi zone public hospitals. The study population comprised all healthcare workers who were selected from these institutions who had been working for at least six months and were directly involved in patient care during the data collection period.

### 2.3. Inclusion and Exclusion Criteria

Healthcare workers aged 18 years or older, with at least six months of work experience, who were directly involved in patient care and provided voluntary informed consent to participate were included in the study. Healthcare workers who were on sick leave, annual leave, or maternity leave during the data collection period were excluded from the study.

### 2.4. Sample Size Determination

The sample size was determined using the single population proportion formula by assuming a proportion (p) of 50% (since no previous study on MVD in Ethiopia is available), a marginal error (d) of 5% (0.05), and a confidence level (CI) of 95% corresponding to Z = 1.96.

$$n=\frac{\left(Z\alpha /2{)}^{2}P(1-P\right)}{d^{2}}\to n=\frac{\left(1.96{)}^{2}(0.50\times 0.50\right)}{(0.05{)}^{2}}=384$$

After adding a 5% non-response rate, the final sample size became 404.

### 2.5. Sampling Procedure and Technique

The list of HCWs was obtained from hospitals’ human resource management offices, and used as a sample frame. A simple random sampling technique (lottery method) was used to select the study participants. All HCWs had an equal chance of being selected.

### 2.6. Operational Definitions

Healthcare worker: This included clinical professionals (physicians, nurses, midwives, and pharmacists) and paraprofessionals (laboratory technicians and anesthetists) directly involved in patient care within the hospital setting [16].

Due to the absence of an absolute cut-off score for knowledge, attitude, and preparedness specific to this context and population, the mean score was chosen as the most reliable way to differentiate between the participants of our study.

Good knowledge: A healthcare worker who scored above the mean value of the total knowledge of 28 items [17].

Poor knowledge: A healthcare worker who scored equal to or below the mean value of the total knowledge of 28 items.

Positive attitude: A healthcare worker who scored above the mean value of the total attitude items.

Negative attitude: A healthcare worker who scored equal to or below the mean of the total attitude out of 27 items.

Adequate preparedness: A healthcare worker who scored above the mean value of the total preparedness of 9 items [18].

Inadequate preparedness: A healthcare worker who scored equal to or below the mean value of the total preparedness questions.

### 2.7. Data Collection Tools and Procedures

The data were collected using a structured, pre-tested, self-administered questionnaire adopted from previous similar studies [14,17]. The tool consisted of the following sections: socio-demographic characteristics of the participants, knowledge and attitude toward Marburg virus, and preparedness of HCWs to prevent MVD. There were 28 questions in the knowledge section and 9 questions in the attitude part. For the knowledge domain, correct answers were given a score of 1, and incorrect answers were given a score of zero. The scores were added together to give the total knowledge score that ranged from 0 to 28, where a high score indicates good knowledge. Regarding attitude, the question was scored on a 3-point Likert scale (disagree, neutral, and agree). The response was given a 1 for disagree, 2 for neutral, and 3 for agree. The scores were added together to give an overall score of 9–27, where a higher score indicates a more positive attitude toward Marburg virus infection preventive measures. The preparedness section had 9 questions (Yes or No) with a score which ranged from 0 to 9.

### 2.8. Data Quality Assurance Technique

Data quality was maintained through a multi-step approach. Pre-testing was done on 5% of the study sample at Finoteselam General Hospital among healthcare workers to assess the questionnaire’s clarity, flow, and consistency. Adjustments, such as wording revisions, were made based on the results obtained from it. Two BSc degree holder data collectors and one supervisor (MPH) received intensive one-day training on the study objectives, ethical issues, and data collection procedures. The principal investigator and supervisor conducted day-to-day follow-ups throughout the data collection period to ensure the completeness and consistency of each questionnaire. The necessary feedback was given to data collectors before leaving the data collection site on the same day. Furthermore, data were cleaned and coded manually before entry to ensure accuracy. Reliability testing was conducted using Cronbach’s alpha, yielding a coefficient of 0.77 for knowledge, 0.74 for attitude, and 0.79 for preparedness which was considered acceptable for internal consistency. All computed coefficients exceeded the standard threshold of 0.70, confirming that the measurement tools were reliable for this study population.

### 2.9. Data Management and Analysis

The collected data were checked and reviewed for completeness, coded, cleaned, edited, and entered into EpiData version 4.6 and were exported to SPSS version 25 for analysis. Data are summarized using proportions for categorical variables and means with standard deviations for continuous variables. The internal consistency of the scales for knowledge, attitude, and preparedness was verified using Cronbach’s alpha. The data are presented using texts and tables.

### 2.10. Ethical Consideration

This research was conducted in accordance with Injibara University’s research ethics guidelines and national and international standards. Ethical clearance was obtained from the Institutional Research Ethics Review Committee (IRERC) of Injibara University on 26 December 2026 (Reference: IU/IRERC/1056/2025). A formal written letter was provided for the respective hospitals, and formal permission was obtained from administrative bodies. Written informed consent was obtained from all participants after explaining the study purpose, procedure, risks, and benefits. Also, they were free to withdraw consent and discontinue participation. All data were anonymized and stored securely. The scholars maintained the ethical principles to ensure the dignity, right, and safety of all participants throughout the study process. This study was conducted in accordance with the Declaration of Helsinki.

## 3. Result

### 3.1. Characteristics of the Study Participants

A total of 394 healthcare workers participated in this study with a response rate of 97.5%. The mean age was 32.9 years (SD ± 4.87), with the majority—305 (77.4%)—falling within the 21–30 years age range. More than half of the participants were male—217 (55.1%)—and married (94.2%). Nurses constituted the largest professional group (52.5%), followed by physicians with 55 (14%), and midwives with 49 (12.4%). Regarding educational background, 204 (51.8%) held a first degree. Three-quarters of the participants—296 (75.1%)—had less than 10 years of work experience.

Regarding information access, nearly all participants—388 (98.5%)—reported awareness of Marburg virus disease (MVD). The primary sources of information were social media, for 372 participants (94.4%), and television (89.3%), whereas medical books and formal study materials were utilized by only 9.1% of respondents (Table 1).

### 3.2. Knowledge About Marburg Virus Disease

The results revealed that nearly all of the participants—388 (98.5%)—had heard about Marburg virus disease and knew about the outbreak in Ethiopia, while 351 (89%) correctly identified MVD is a viral infection, and 86% recognized its fatality. Although 71.8% correctly identified bats as the reservoir and 98.7% were aware of human-to-human transmission potential, only 69.3% recognized the zoonotic animal-to-human transmission route. Regarding clinical aspects, 74.6% knew the incubation period (2–21 days). Furthermore, 66.8% knew diagnostic modalities, and 80.5% were aware that symptomatic management involves antipyretics and IV fluids. The proportion of participants with good overall knowledge was 47.7% (95% CI: 42.78–52.62%) (Supplementary Table S1).

### 3.3. Attitude Towards Marburg Virus Disease

The results indicated that participants had strong perceptions of risk and systemic concerns, along with a considerable demand for additional education. The study revealed that the majority, 357 (90.6%), of the participants wanted to learn more about Marburg virus disease. Of the participants, 366 (92.9%) perceived themselves as being at risk, and 92.4% of HCWs believed they were prone to Marburg virus infection. Most of the participants, 340 (86.3%), thought that Marburg virus disease could add a new burden to the healthcare system of affected countries, and around 74.1% of participants agreed that the infection control policy of each hospital was inadequate. The mean attitude score was 22.63 ± 2.59 (Table 2). Overall, 61.2% (95% CI: 56.4–66.0%) of participants demonstrated a positive attitude towards MVD prevention and control, with a mean attitude score of 22.63 (SD ± 2.59).

### 3.4. Preparedness for Marburg Virus Disease Outbreak

The study showed that only 93 (23.6%) participants had ever received training on emergency preparedness; of those, only 58 (62.4%) reported that the training included MVD preparedness. Consequently, the majority of the participants, 301 (76.4%), expressed strong interest in receiving emergency preparedness training (Table 3). The overall preparedness of healthcare workers to prevent and control a Marburg virus outbreak was 20.3% (95% CI: 16.34–24.26%). The mean score of preparedness was 3.92 ± 1.24.

## 4. Discussion

This study assessed the knowledge, attitude, and preparedness towards MVD among HCWs in Awi Zone public hospitals, northwest Ethiopia. The findings provided critical insights into the frontline healthcare workers’ readiness in a region potentially at risk following the declared outbreak in southern Ethiopia. This study revealed a critical disconnect among healthcare workers (HCWs) in the Awi Zone: while awareness of Marburg virus disease (MVD) is nearly universal and perceived risk is high, functional preparedness remains critically low.

In this study nearly half (47.7%) of HCWs demonstrated good knowledge of MVD. This result is similar to a study done in Uganda, where 52% of HCWs had adequate knowledge of Marburg virus disease [19]. However, it is notably lower than study findings reported from India, where 68.5% of HCWs demonstrated good knowledge of MVD [20]. This discrepancy may be attributed to differences in prior outbreak exposure, availability of training opportunities, health education infrastructure, and access to updated health information across the regions. Unlike India, where continuous medical education is more structured and training about emergency diseases is provided, HCWs in the present study relied heavily on mass media (94.4%) for information. While media raises awareness, it may not provide the depth of understanding required for clinical competence, potentially explaining the suboptimal knowledge observed. On the contrary, the finding of this study is higher than the study reported in Egypt, which revealed that only 12.5% of participants demonstrated good knowledge about Marburg virus infection [21]. This variation might be due to the temporal context of the current study, conducted immediately after a confirmed national outbreak (November 2025), which likely heightened information dissemination compared to pre-outbreak periods in other regions.

Regarding attitudes, 61.2% of HCWs demonstrated a positive attitude toward MVD prevention and control. The finding is consistent with studies conducted in Uganda (60.5%) [19] and India (60.5%) [20], where a similar proportion of healthcare workers reported a positive attitude towards the control and prevention of Marburg virus disease, despite variation across professional groups. However, the level of positive attitude observed in this study is higher than the study reported in Egypt, which revealed that only 15% of nurses had a positive attitude towards Marburg virus infection [21]. The high positive attitude in the study area, driven largely by the fact that 92.9% of participants perceived themselves as being at risk, suggests that the recent national outbreak successfully instilled a sense of vulnerability among HCWs. This result is comparable to the studies done in Uganda (60.5%) [19] and India (60.5%) [20], where HCWs recognized their vulnerability but debated systemic readiness.

In spite of relatively positive knowledge and attitudes, preparedness for an MVD outbreak remained low. Only 20.3% of HCWs felt prepared for an MVD outbreak. This is significantly lower than reports from a Nigerian referral center, where 44% of doctors and nurses felt prepared for an MVD response [11]. We critically interpret this discrepancy not merely as a lack of knowledge, but as a structural deficit. Similarly, the finding of this study is lower than a study conducted in Western Africa [18]. In the present study, preparedness gaps were evident, including limited training coverage (23.6%), low participation in emergency drills (13.2%), and inadequate availability of isolation facilities (14.9%). This might be due to the recurrent outbreaks of MDV in western Africa; as a result, timely and concrete preparedness has been done through proper surveillance, especially in high-risk areas, as well as proven training for health workers and routine updates on the co-occurrence of multiple outbreaks of deadly viruses and the high potential of the emergence of MARV. This aligns with WHO reports, which showed that essential IPC infrastructure for the management of VHF, including trained personnel, is not available in many African health facilities [12].

This low preparedness level observed here is reflected in what occurred during the Marburg epidemic in Angola in 2005, where a lack of adequate IPC for VHF measures led to many HCWs becoming infected [22]. This underscores the urgent need for investment in health system readiness, especially in regions with recent outbreaks.

### 4.1. Implications

The findings of this study reveal moderate knowledge, a concerning attitude–risk–awareness mismatch, and critically low preparedness, all of which carry immediate and significant implications for clinical practice, health system management, and public health policy in Ethiopia and in similar contexts. The poor preparedness score (20.3%) with a high perception of insufficient IPC policies (74.1%) is an urgent warning for administrators of hospitals as well as the Health Department of Awi Zone. The message for the Regional Health Bureau and Ministry of Health is to develop and distribute standardized, updated training modules on high-threat pathogens like MVD, through integration into compulsory in-service training. In clinical practice, the knowledge gaps imply that individual clinical suspicion for MVD must be heightened. Healthcare workers should have a low threshold for isolating patients with non-specific febrile illness and travel history, adhering strictly to IPC measures, irrespective of confirmed diagnosis.

### 4.2. Strengths and Limitations

This study had strengths, including a robust sample size, high response rate, and multicenter design. Despite this, it had limitations. First, the cross-sectional design precludes the establishment of causal relationships between the assessed variables. Second, the study is susceptible to measurement biases. The primary outcome measures (knowledge, attitude, and preparedness) were self-reported, making them vulnerable to social desirability bias. Third, the operationalization of the outcome variables relied on arbitrary cut-off points. The classification of participants as having “good” or “poor” knowledge, or “positive” or “negative” attitudes, was determined using mean scores and percentiles specific to this study sample. Finally, the participants were recruited exclusively from public hospitals which also limits the generalizability of our findings to private facilities or primary care settings.

### 4.3. Conclusions

This study indicates moderate knowledge and positive attitudes but critically low preparedness levels for Marburg virus disease prevention and control among healthcare workers in Awi Zone public hospitals, northwest Ethiopia. Despite the fact that HCWs were aware of MVD and perceived it as a threat, systemic and individual preparedness remained inadequate to mount an effective outbreak response. In order to overcome these challenges, healthcare workers must engage in MVD training, as well as advocate for better IPC resources and protocols within their facilities to improve individual as well as professional safety. Regional Health Bureau and hospital administrators should develop and update MVD-specific emergency preparedness and response plans, conduct simulation drills, providing regular in-service training about MVD, and ensure the availability of IPC materials. Public Health Authorities should strengthen surveillance, use official channels to disseminate accurate information, and provide regular competency-based training for healthcare workers. In the future, researchers should conduct qualitative research to explore contextual barriers to preparedness and the effectiveness of training interventions.

## Figures and Tables

**Table 1:** Baseline characteristics of participating healthcare workers of public hospitals in the Awi Zone, northwest Ethiopia, in 2026 (*n* = 394).

Variable	Category	Frequency	Percentage
Age	21–30 years	305	77.4
	31–40 years	74	18.8
	41–50 years	15	3.8
Sex	Male	217	55.1
	Female	177	44.9
Marital status	Married	371	94.2
	Single	23	5.8
Educational status	Diploma	120	30.4
	First degree	204	51.8
	Masters and above	70	17.8
Profession	Nurse (all types)	207	52.5
	Midwives	49	12.4
	Physicians	55	14.0
	Others *	83	21.1
Work experience (years)	<10 years	296	75.1
	≥10 years	98	24.9
Monthly salary (ETB)	<20,000	268	68
	20,000–25,000	78	19.8
	>25,000	48	12.2
Access to information about Marburg virus disease	Yes	388	98.5
	No	6	1.5
Source of information †	Social media	372	94.4
	Television	352	89.3
	Colleague	187	47.5
	Medical books/during study	36	9.1

Others *: laboratory workers, pharmacists, anesthetists, psychiatrists, public health officers & IESOPS. † Multiple responses possible.

**Table 2:** Attitude of healthcare workers regarding Marburg virus outbreaks, in Awi Zone Public Hospitals, northwest Ethiopia, in 2026 (*n* = 394).

Statements	Disagree *n* (%)	Neutral *n* (%)	Agree N (%)
Interested in learning more about Marburg virus disease	32 (8.1)	5 (1.3)	357 (90.6)
Interested in learning more about the epidemiology of new emerging diseases	61 (15.5)	6 (1.5)	327 (83)
Consider self to be at risk	20 (5.1)	8 (2)	366 (92.9)
Health workers are prone to having Marburg virus disease	18 (4.6)	12 (3)	364 (92.4)
Marburg virus disease can add a new burden on the healthcare system of affected countries	20 (5.10)	34 (8.6)	340 (86.3)
Prevent MVD spread is possible	37 (9.4)	44 (11.2)	313 (79.4)
I have reservations/doubts about the virus’s genesis as it is now explained	153 (38.8)	102 (25.9)	139 (35.3)
There is no risk in living with MVD patient	321 (81.5)	34 (8.6)	39 (9.90)
Infection control policy of the hospital is inadequate	49 (12.4)	53 (13.5)	292 (74.1)
Mean score (±SD)	22.63 ± 2.59		

**Table 3:** Preparedness of healthcare workers for Marburg virus outbreak in Awi Zone public hospitals, northwest Ethiopia, in 2026 (*n* = 394).

Preparedness Questions	Category	Frequency	Percentage
Received training on emergency preparedness	Yes	93	23.6
	No	301	76.4
Desire to receive training on emergency preparedness	Yes	301	76.4
	No	93	23.6
Training included MVD preparedness	Yes	58	62.4
	No	35	37.6
Emergency drill done	Yes	52	13.2
	No	342	86.3
Emergency plan for Marburg virus exist	Yes	42	10.7
	No	352	89.3
Health workers improvise IPC materials	Yes	68	17.3
	No	326	82.7
Isolation space is available for suspected cases	Yes	58	14.9
	No	336	85.1
Experienced any case of transmission among health workers	Yes	54	13.7
	No	340	86.3
Adequate measures currently being implemented to prevent future transmission	Yes	46	11.7
	No	348	88.3
Mean score (±SD)	3.92 ± 1.24		

## Data Availability

Data are available from the corresponding author upon reasonable request.

## Supplementary Materials

The following supporting information can be downloaded at: https://www.mdpi.com/article/10.3390/tropicalmed11050125/s1, Table S1: Knowledge of Healthcare workers about Marburg virus in Awi Zone Public Hospitals, Northwest Ethiopia, 2026 (*n* = 394).

## Abbreviations

HCWs	Healthcare Workers
IPC	Infection Prevention and Control
MARV	Marburg Virus
MVD	Marburg Virus Disease
PPE	Personal Protective Equipment
WHO	World Health Organization

## References

[1] Francis D.L., Reddy S.S.P., Logaranjani A., Karthikeyan S.S.S., Rathi M. (2025). Marburg Virus Disease: Pathophysiology, Diagnostic Challenges, and Global Health Preparedness Strategies. Ann. Glob. Health.

[2] Srivastava S., Sharma D., Kumar S., Sharma A., Rijal R., Asija A., Adhikari S., Rustagi S., Sah S., Al-Qaim Z.H. (2023). Emergence of Marburg virus: A global perspective on fatal outbreaks and clinical challenges. Front. Microbiol..

[3] Asad A., Aamir A., Qureshi N.E., Bhimani S., Jatoi N.N., Batra S., Ochani R.K., Abbasi M.K., Tariq M.A., Diwan M.N. (2020). Past and current advances in Marburg virus disease: A review. Infez. Med..

[4] Amman B.R., Bird B.H., Bakarr I.A., Bangura J., Schuh A.J., Johnny J., Sealy T.K., Conteh I., Koroma A.H., Foday I. (2020). Isolation of Angola-like Marburg virus from Egyptian rousette bats from West Africa. Nat. Commun..

[5] World Health Organization (2023). WHO Framework for Meaningful Engagement of People Living with Noncommunicable Diseases, and Mental Health and Neurological Conditions.

[6] European Public Health (2025). Murburg viruse disease interim guidance for laburatory specimen collection and management. Biosafty Bio-Secur..

[7] Rugarabamu S., Neel G. (2023). Global Preparedness and Response Strategies for Emerging Viral Hemorrhagic Fevers: Lessons from Past Outbreaks. Epidemiol. Public Health.

[8] Belhadi D., El Baied M., Mulier G., Malvy D., Mentré F., Laouénan C. (2022). The number of cases, mortality and treatments of viral hemorrhagic fevers: A systematic review. PLoS Neglected Trop. Dis..

[9] Raab M., Pfadenhauer L.M., Millimouno T.J., Hoelscher M., Froeschl G. (2020). Knowledge, attitudes and practices towards viral haemorrhagic fevers amongst healthcare workers in urban and rural public healthcare facilities in the N’zérékoré prefecture, Guinea: A cross-sectional study. BMC Public Health.

[10] Pigott D.M., Deshpande A., Letourneau I., Morozoff C., Reiner R.C., Kraemer M.U., Brent S.E., Bogoch I.I., Khan K., Biehl M.H. (2017). Local, national, and regional viral haemorrhagic fever pandemic potential in Africa: A multistage analysis. Lancet.

[11] Adjei G.A., Okai G.A., Agboh H.K.N., Adjare L.O., Dzackah H. (2025). The Marburg virus disease in Ghana: Psychological impact of quarantine on health workers in the Adansi-North district. BMC Psychol..

[12] World Health Organization (2024). Global Report on Infection Prevention and Control 2024.

[13] Oisakede E., Asogun D., Otaigbe O., Iyoriobhe I., Erhieyovwe E., Emorinken A., Nwosu M., Osamudiamen U. (2025). Health literacy and preparedness for outbreak of Marburg Virus Disease among doctors and nurses at a reference treatment centre for viral hemorrhagic diseases in Nigeria. J. Interv. Epidemiol. Public Health.

[14] Oisakede E.O., Asogun D., Otaigbe O., Iyoriobhe I., Erhieyovwe E.O., Emorinken A., Nwosu M., Osamudiamen U.M., Olawade D. (2026). Assessment of healthcare worker preparedness and health literacy for Marburg virus disease in Nigeria: A cross-sectional study. S. Afr. J. Infect. Dis..

[15] Agboh H.N.K., Adjei G.A., Okai G.A., Awotwe C., Ossom B.M., Yarney L. (2024). Infection prevention and control: Qualitative study of the preparedness and response of Christian health Association of Ghana to Marburg virus disease in Ghana. Heliyon.

[16] World Health Organization (2022). WHO Guidance on Research Methods for Health Emergency and Disaster Risk Management.

[17] Bitew G., Sharew M., Belsti Y. (2021). Factors associated with knowledge, attitude, and practice of COVID-19 among health care professional’s working in South Wollo Zone Hospitals, Northeast Ethiopia. SAGE Open Med..

[18] Reuben R.C., Abunike S.A. (2023). Marburg virus disease: The paradox of Nigeria’s preparedness and priority effects in co-epidemics. Bull. Natl. Res. Cent..

[19] Nyakarahuka L., Skjerve E., Nabadda D., Sitali D.C., Mumba C., Mwiine F.N., Lutwama J.J., Balinandi S., Shoemaker T., Kankya C. (2017). Knowledge and attitude towards Ebola and Marburg virus diseases in Uganda using quantitative and participatory epidemiology techniques. PLoS Neglected Trop. Dis..

[20] Mehta V., Negi S., Mathur A., Obulareddy V.T., Shaik R.A., Ahmed M.S., Miraj M. (2024). Knowledge, attitude, and perception about marburg virus in healthcare workers of India. Discov. Public Health.

[21] Hessien Yousef Heggy E., Mohamed Elmansy F., Tawfik Al Manzantia S., Elsayed Awad Negm H., Esmat Mahmoud Khalil H. (2023). Effect of Educational Sessions on Marburg Viral Infection at Fever and Chest Outpatient Clinics in Mansoura Hospitals. Egypt. J. Health Care.

[22] Muvunyi C.M., Ngabonziza J.C.S., Bigirimana N., Ndembi N., Siddig E.E., Kaseya J., Ahmed A. (2024). Evidence-based guidance for one health preparedness, prevention, and response strategies to marburg virus disease outbreaks. Diseases.

