# Supplementary material for: Knowledge, Attitudes, and Preparedness Regarding Marburg Virus Disease Among Healthcare Workers in Awi Zone Public Hospitals, Northwest Ethiopia: A Multicenter Cross-Sectional Study
Source: Trop Med Infect Dis. 2026 May 6;11(5):125. doi: 10.3390/tropicalmed11050125 (PMC13211424; doi:10.3390/tropicalmed11050125)
Supplement: Supplementary file 1 [file tropicalmed-11-00125-s001.zip › tropicalmed-4217328-supplementary.pdf]

Supplementary File

Table S1: Knowledge of Healthcare workers about Marburg virus in Awi Zone Public Hospitals, Northwest Ethiopia, 2026 (n=394)

**Knowledge questions**	**Category**	**Frequency**	**Percentage**
Marburg virus disease is prevalent in Western and Central Africa	Yes	371	94.2
	No	23	5.8
There is/are confirmed human Marburg virus cases in Ethiopia	Yes	378	98.5
	No	6	1.5
Marburg disease is a viral infection	Yes	351	89
	No	41	11
The reservoir is usually bats	Yes	283	71.8
	No	111	28.2
Infection with the organism is usually deadly	Yes	339	86
	No	55	14
Incubation period last from 2 to 21 days	Yes	294	74.6
	No	100	25.4
Marburg is easily transmitted from person to person	Yes	389	98.7
	No	5	1.3
Marburg can be transmitted from animal to person	Yes	273	69.3
	No	121	30.7
Marburg can be transmitted from inanimate objects to person	Yes	380	79.7
	No	14	20.3
Marburg can be transmitted through skin-to-skin contact	Yes	292	74.1
	No	102	25.9
Marburg can be transmitted through blood	Yes	301	76.4
	No	93	23.6
Marburg can be transmitted through seminal/vagina fluid	Yes	294	74.4
	No	100	25.6
Bodies of dead cases constitute a potential hazard	Yes	333	84.5
	No	61	15.5
International travel is the main source of imported cases of Marburg	Yes	300	76.1
	No	94	23.9
Marburg and Ebola have almost similar signs and symptoms	Yes	312	79.2
	No	82	20.8
Marburg virus is less contagious than Ebola virus	Yes	235	59.6
	No	159	40.4
The first phase, known as the generalized phase, lasts from days one to four and is characterized by an abrupt onset with nonspecific, flu-like symptoms, including high fever (typically between 39 and 40 ◦C), severe headache, chills, myalgia, prostration, and malaise	Yes	283	71.8
	No	111	28.2
The early phase, spans from 5 to 13 days: Anorexia, abdominal discomfort, severe nausea, vomiting, diarrhea, maculopapular rash, and symptoms of hemorrhagic fever (petechiae, mucosal, and gastrointestinal bleeding, hemorrhage from venipuncture sites)	Yes	277	70.3
	No	117	29.7
The final phase, the convalescence phase, begins after day 3days: Neurological symptoms may present(disorientation, agitation, seizures, and coma), recovery symptoms	Yes	250	63.5
	No	144	36.5

Hand sanitizers are important in preventing Marburg disease	Yes	279	70.5
	No	115	29.5
(RT-PCR) assay, IgG and IgM antibodies with ELISA &Antigen detection tests are means of Dx of MVI	Yes	263	66.8
	No	131	33.2
One management option for symptomatic Marburg disease patients is to use Antipyretics, Iv fluids,	Yes	316	80.5
	No	78	19.5
Antiviral drugs are required in the management of patients with Marburg disease	Yes	184	46.7
	No	210	53.3
Antibiotics are required in the management of patients with Marburg disease	Yes	47	11.9
	No	347	88.1
There is a specific vaccine for Marburg disease	Yes	29	7.4
	No	365	92.6
There is a specific treatment for Marburg disease	Yes	78	19.2
	No	316	80.8
Environmental sanitation protects from Marburg virus infection	Yes	274	69.5
	No	120	30.5
Safe sex protects from infection	Yes	269	68.3
	No	125	31.7
Barrier nursing protects from infection	Yes	323	82
	No	71	18
Mean score (± SD)	16.5±4.45		
